# The costs and benefits of research grant funding peer review

**DOI:** 10.12688/f1000research.176989.1

**Published:** 2026-04-16

**Authors:** Alexandra Pollitt, Clare Taylor, Niall Sreenan, Jonathan Grant

**Affiliations:** 1 Policy Institute, King's College London, London, England, UK; 2 University of St Andrews School of Modern Languages, St Andrews, Scotland, UK; 3 King Abdullah University of Science and Technology, Thuwal, Makkah Province, Saudi Arabia

**Keywords:** Grant peer review, Research funding efficiency, Transaction costs, Peer review burden, Research funding policy, Cost–benefit analysis, Academic labour, Research evaluation, Meta science

## Abstract

Understanding the costs of developing, writing, reviewing, and deciding on research proposals is a critical prerequisite for understanding the efficiency of funding decisions. The objective of this study was to develop estimates for cost and transaction cost – the ratio of cost to funding – for a sample of different funding schemes administered by two funders. The perceived benefits of the peer-review funding process to applicants, reviewers, and panelists were also explored. A total of 12,617 individuals were surveyed between April 2022 and June 2023, with an overall response rate of 11%. The study estimated that the cost of grant and fellowship application processes is 13% of the value of the grant and that 89% of those costs are borne by applicants. This would suggest that any policy to increase the efficiency of grant funding (beyond increasing success rates) should focus on university and research institute practices rather than on research funders.

## 1. Introduction

The cost of developing, writing, reviewing, and deciding on research proposals is an under-researched but critical prerequisite for understanding the efficiency of funding decisions. This is increasingly a priority issue for governments, with former UK Prime Minister Johnson stating in 2019 his wish to reduce funding red-tape “to ensure brilliant scientists are able to spend as much time as possible creating new ideas, not filling in unnecessary forms.” ^1^ This commitment was supported by the publication of a policy paper by the UK Government on ‘Reducing bureaucratic burden in research, innovation and higher education’ ( BEIS and DfE, 2020) and an ‘Independent Review of Research Bureaucracy’ led by the Vice-Chancellor of the University of Birmingham, Prof Alan Tickell ( Tickell, 2022). The Tickell Review made six recommendations, including the need for “Proportionate funding applications: ask for less information at the start of the process and only ask for more as the chances of success get higher”. The UK Government responded to the Tickell Review in February 2024 ( DSIT, 2024), making a number of ‘key commitments,’ as set out in Box A. In the foreword to this document, the Secretary of State for Science, Innovation and Technology, Michelle Donelan, echoed Johnson’s comments five years previous, saying, “If we keep tying the hands of our brilliant scientists and world-leading institutions with layer upon layer of endless bureaucracy, we simply will not fulfil our potential as a nation”.

Box A: **The key commitments of UK government to reduce research bureaucracy in the grant/fellowship application process.**

• Funders will streamline and experiment with new application processes, taking proportionate approaches to reducing administrative burdens.
• Funders will increase standardisation in their application processes and will work together to harmonise their approaches, as far as is practicable.
• Assessment processes will be reviewed and new methods trialled.
• Funders will ensure that innovation in the application and assessment processes supports equality, diversity, and inclusion.
• The requirement for letters of support will be removed, or if retained because it is a critical part of the assessment process, the information required will be significantly reduced.

Whilst this focus on ‘red tape’ may seem political, Chalmers et al. (2014) suggested that 85% of medical research funding is wasted. This waste occurs throughout the research pipeline; however, two of the four steps identified by Chalmers et al. – asking the right research question and using the right methods to address the research question – are key to peer review. Based on the Chalmers model, getting these two questions wrong could account for up to half of the estimated waste.

Peer review also plays an important role in funding allocation and may bring benefits. For example, the peer review process could strengthen a grant, allowing the honing of both research questions and methods, and as such, reduce waste and bring value to the scientific process. However, our ability to assess these benefits and burdens is hampered by our limited understanding and lack of empirical, comparative evidence on how peer review processes work ( de Vrieze, 2017; Guthrie et al., 2018; Guthrie, 2019).

The key objective of this study is to develop estimates for the cost and transaction costs – the ratio of cost to funding – for a sample of different funding schemes administered by two funders, and to break down those cost estimates by various characteristics and ‘cost drivers’. Simultaneously, the perceived benefit of the peer review funding process to applicants, reviewers, and panelists was explored. As discussed at the end of this paper, in the future, it should prove possible to monetize the value of the benefits of peer review to allow a cost-benefit assessment; however, this is not feasible at present, and thus it is necessary to make a judgement of whether perceived value, as identified by participants, justifies costs.

## 2. What is known about the costs and benefits of grant peer review

Hinrichs-Krapels and Grant (2016) proposed an exploratory framework to evaluate how research is assessed, based around the ‘3Es’ of effectiveness, efficiency and equity. They looked at both *ex-ante* and *ex-post* research assessments, but here the focus is on the *ex-ante *, that is, the peer review process that supports decisions about which research to fund. In their article, Hinrichs-Krapels and Grant argued that most research evaluations have focused on the effectiveness of research through the assessment of research outputs and impacts by using, for example, bibliometrics or, more recently, impact case studies, Overton, *Altmetrics* and *Researchfish.* They noted that similar tools are not yet available to systematically evaluate the efficiency and equity of research assessments.

This study addresses this gap in our knowledge of efficiency by developing our understanding of the costs and benefits (or value) of peer review. Processes such as peer review have been noted to incur substantial costs for upholding quality ( Wessely, 1998) and questions have been raised about its cost-effectiveness ( Godlee and Jefferson, 1999).

In 1948, Leo Szilard, a Hungarian-German-American physicist (who patented the idea of a nuclear reactor in 1934) envisaged the future and fictional Mark Gable Foundation for the “retardation of scientific progress” ( Szilard, 1961). ^2^ In a satirical piece, he describes a peer review process for the new foundation based on the National Science Foundation. As summarized in Box B, when asked how this would “retard the progress of science,” he explained that the time required to prepare and review an application, and the chance of its success, meant that a scientist’s time would consist solely of writing or reviewing applications, with no time left for actually doing the research itself.

Box B: **Extract from the ‘The Mark Gable Foundation’ in *‘The Voice of the Dolphins and Other Stories’* ( Szilard, 1961).**

“Would you intend to do anything for the advancement of science?” I asked.

“No,” Mark Gable said. “I believe scientific progress is too fast as it is.”

“I share your feeling about this point,” I said with the fervor of conviction, “but then why not do something about the retardation of scientific progress?”

“That I would very much like to do,” Mark Gable said, “but how do I go about it?”

“Well,” I said, “I think that shouldn’t be very difficult. As a matter of fact, I think it would be quite easy. You could set up a foundation, with an annual endowment of thirty million dollars. Research workers in need of funds could apply for grants, if they could make out a convincing case. Have ten committees, each composed of twelve scientists, appointed to pass on these applications. Take the most active scientists out of the laboratory and make them members of these committees. And the very best men in the field should be appointed as chairmen at salaries of fifty thousand dollars each. …”

“I think you had better explain to Mr. Gable why this foundation would in fact retard the progress of science,” said a bespectacled young man sitting at the far end of the table, whose name I didn’t get at the time of introduction.

“It should be obvious,” I said. “First of all, the best scientists would be removed from their laboratories and kept busy on committees passing on applications for funds. Secondly, the scientific workers in need of funds would concentrate on problems which were considered promising and were pretty certain to lead to publishable results. For a few years there might be a great increase in scientific output; but by going after the obvious, pretty soon science would dry out. Science would become something like a parlor game.”

Despite these long-standing concerns about the burden, cost, and cost-effectiveness of peer review, surprisingly little research has been conducted on this topic, as summarized in Table 1. The most significant study in the UK is the 2006 report by Research Councils UK (RCUK) ( RCUK, 2006) on the efficiency and effectiveness of peer review. This study looked at the cost of preparing proposals, the cost of peer review, the administrative cost to the Research Councils, and the cost of preparing the end of grant reports and concluded that this was equivalent to £196 m per year (in 2006 money), equivalent to 13% of the total funding awarded. Non-RCUK costs were based on interviews with 93 researchers and 27 administrators.

**Table 1.** Summary of current literature on assessing the costs of peer review.

Study	Transaction cost	Method	References
UK Research Councils	13%	Includes RCUK internal costs, preparation and submission and peer review. Non-RCUK costs based on interviews.	RCUK (2006)
UK Funders	c5%	Based solely on administrative costs of funders, taken from annual reports.	Morton et al. (2012)
New Zealand Marsden Fund	20–35%	Based on report by the Royal Society of New Zealand, which provides a ‘best estimate’ with no clarity on how this was derived.	Gluckman (2012); Weston and Gush (2012)
Australian NHMRC	14%	Survey of researcher time.	Herbert et al. (2013)
UK Research Excellence Framework (2014)	2.4%	Costs analysis based on various sources including interviews and surveys.	Technopolis (2015)
UK Research Excellence Framework (2021)	3–4%	Costs analysis based on various sources including interviews and surveys.	Technopolis (2023)

This prompted a small number of other studies. For example, Morton et al. (2012) compared administration costs as a percentage of the total budget for UK funders such as the Wellcome Trust, Medical Research Council, and Department for International Development, which ranged from 2.8 to 7.1% of the total budget. However, this is clearly a crude measure: first, these funders do more than administer grants; second, as shown in the RCUK report ( RCUK, 2006) and subsequent studies, the majority of costs are external to the funding agency, either through the time applicants take to prepare their proposals or through the review of the grant by referees or panel members.

Gluckman (2012), then Chief Science Advisor to the Prime Minister of New Zealand, and Weston and Gush (2012) included these additional costs when they examined the investigator-initiated Marsden Fund and estimated the transaction cost to be as high as 20–35%, with the majority of costs falling on applicants. The direct administrative costs of operating the grant process were less than 3% of the fund size. They further concluded that the time spent writing proposals represents over 80% of the total cost, with three-quarters of that spent on first-stage proposals. International reviewers and panelists accounted for 10% of the total cost (which is a significant burden on the small number of people who are called upon in these roles). One important point that Gluckman (2012) notes is that for unsuccessful applicants, this may not be time that is entirely wasted; there are clear intangible benefits from researching and clarifying ideas, building networks, and the possibility of using those applications to access alternative funding.

Herbert et al. (2013) estimated the applicant burden for the Australian National Health and Medical Research Council (NHMRC). However, their focus was the time researchers spent preparing their applications and thus excluded the administrative costs of the funding agency and the time spent by peer reviewers and panelists. Nevertheless, they concluded that the transaction cost (for applicants) was 14% based on 285 completed surveys. This estimate was derived from a sample of researchers who submitted a grant proposal to NHMRC in March 2012. Each researcher was asked if they were the lead researcher, how much time they spent (in days), and whether the proposal was new or a resubmission. They were also asked about their previous experience with the peer-review system, as an expert panel member or external peer reviewer, and their salary to estimate the financial costs of preparing proposals.

More recently in the UK there have been efforts to measure the costs of the 2014 and 2021 Research Excellence Framework (REF). The total transaction costs for both the universities and funding councils for REF2014 were £246 m or 2.4% of the total money allocated ( Technopolis, 2015) and for REF2021, £471 m or 3–4% of the total allocated funding ( Technopolis, 2023). The most recent estimate comprises approximately £454 m (96%) in costs to the higher education community and around £17 m (4%) in costs for funding bodies. Of that £454 m, Technopolis estimated the costs to be around £430 m for the submission process and £24 m for panelists.

Finally, in a recent study of the European Union’s Horizon 2020 research and innovation program, Rodriguez-Rincon et al. (2022) found that applicants spend 27 days on a single stage and 43 days on a multi-stage application (in cases where the application is subject to interview). These findings were based on a survey of the applicants and reviewers for Horizon 2020. Similar to Gluckman (2012) and Weston and Gash (2012), Rodriguez-Rincon et al. (2022) found that most time is spent on writing applications – 85% of the time is spent on writing for single-stage applications, rising to 91% for multi-stage applications. Reviewers spent approximately four to six days in total, collectively, reviewing each proposal. This increased to 5.5–7.5 days for processes that included an interview. Note that Rodriguez-Rincon et al. (2022) did not monetize these estimates, meaning that transaction costs could not be calculated (and thus, this study is excluded from Table 1).

As already noted, costs are only one consideration in the allocation of funding, and it is useful to be aware of the benefits that review processes produce, both in terms of their effectiveness for optimizing the allocation of funding and any wider benefits they confer on the research system and the various actors involved (as noted by Gluckman, 2012). Assessing the effectiveness of allocation in supporting the ‘best’ research would rely on the ability to conduct robust, comparable assessment of research outcomes and impact, in itself a major challenge, which could then be linked back to specific aspects of the review process. To the best of our knowledge, this is not something that has been explored systematically beyond, for example, bibliometric studies focusing solely on the immediate outputs of research (e.g., Ayoubi et al., 2019; Robitaille et al., 2016).

A small, mostly qualitative, literature has explored some of the additional benefits of peer review processes. These studies suggest that, in addition to the primary goal of obtaining research funding, applicants might benefit from refining their thinking, generating new ideas, building collaborations, and considering the ‘bigger picture’ in their research areas ( Ayoubi et al., 2019; Irwin et al., 2013; von Hippel & von Hippel, 2015), as well as being stimulated to think differently by the remit of a specific request for proposals. Grant reviewers and panel members can also benefit from participating in review processes, for example by improving their own ‘grant(wo)manship,’ having early sight of new findings and methodological developments, capitalizing on opportunities for networking, enhancing their CVs and fulfilling a perceived duty to ‘give back to the scientific community’ ( Gallo et al., 2020; Irwin et al., 2013; Schroter et al., 2010). Funders may also gain additional benefits from peer review, beyond simply allocating funding. While the literature is limited in this regard, they may benefit from, for example, building confidence and trust in their allocation processes (e.g., Gluckman et al., 2017), maintaining relationships with academic communities via panels/reviewers, and keeping up-to-date with research developments.

Several conclusions can be drawn from this brief review of the literature. First, there is a lack of consistency in how peer review costs are calculated and expressed. Second, there are different sources and stages in which and when costs and benefits accrue. For example, in the literature, there seem to be three key sources of costs: the cost to the applicant in preparing a proposal, the cost to the funding agency in administering the proposal, and the cost to the reviewers in either being referees or panelists in assessing the proposal. Similarly, the same stakeholders may accrue benefits as noted above. The stages are less clear but broadly consist of preparation, review, response, and decision-making. Finally, cost is only one consideration when assessing peer review: there is a legitimate argument that the focus should be on cost-effectiveness (i.e., the ‘quality’ of the peer review process) and appropriateness of decision-making (i.e., taking into account risk-appetite, size, and value of funding call).

## 3. Methods

The primary objective of the project was to estimate the monetized costs of the peer review process for a sample of research grant or fellowship schemes, and to balance that against the value derived from the process for the applicants, reviewers, panel members, and Research Council staff. To meet this objective, a mixed-method approach was adopted, employing both a survey and key informant interviews. The approach is summarized below, with more details provided in Supplementary Materials Annex A.

### Selecting research funding programmes

The focus of the study was on researchers making grant or fellowship applications to the UK’s Medical Research Council (MRC) and Engineering and Physical Sciences Research Council (EPSRC), both part of UK Research & Innovation (UKRI). These two disciplinary-focused funders were chosen because: a) the MRC funded, via a peer-reviewed research grant, the study; b) the EPSRC were interested in the topic, had a different disciplinary focus from the MRC, and had a different approach to peer review of research. That is, the MRC operated with set deadlines for submission to specified panels, whereas the EPSRC had no deadlines with a collegiate body of reviewers convened for panels at regular intervals. In discussions with both funders, four funding schemes with varying objectives were selected:

• **MRC Developmental Pathway Funding Scheme (DPFS)** is for “academically-led translational projects that aim to either: improve prevention, diagnosis, prognosis, or treatment of significant health needs; and develop research tools that increase the efficiency of developing interventions. All diseases and interventions are eligible for support. You can also address global health issues. Your project can start and finish at any stage on the developmental pathway from early development, through pre-clinical refinement and testing to early-phase clinical studies and trials (up to phase 2a).” ^3^
• **MRC Research Grants** “are suitable for focused research projects that may be short- or long-term in nature. In addition, they can be used to support method development or development and continuation of research facilities, and may involve more than one research group or institution. A research grant can be awarded for any period of up to five years.” ^4^
• **EPSRC Standard Research Grants** “are for researchers at UK higher education institutions … [The EPSRC encourages] collaboration with other researchers, industry, the public sector and other relevant partners; high-risk and high-return research into new concepts and techniques. Projects can range in size from small, short-term grants to multi-million-pound research programmes lasting several years. There is no limit on the size of the grant or length of the project.” ^5^
• **EPSRC Fellowships** “support talented and ambitious researchers to deliver research excellence and lead our research base in evolving towards a modern working culture ... Fellowships provide applicants with the flexibility and freedom to design a package that fits their career ambitions, research needs and personal development.” ^6^

These schemes were chosen based on reviewing data on the number of applicants, diversity of objectives and processes, timing of any call for proposals, and discussions with both funders. The intention was that they comprised a balance of ‘standard’ grant mechanisms, fellowships and ‘one-off’ schemes that would capture a variety of methods, processes, forms of assessment, and benefits of grant funding peer review.

### Surveying applicants, reviewers and panel members

**Figure 1.**
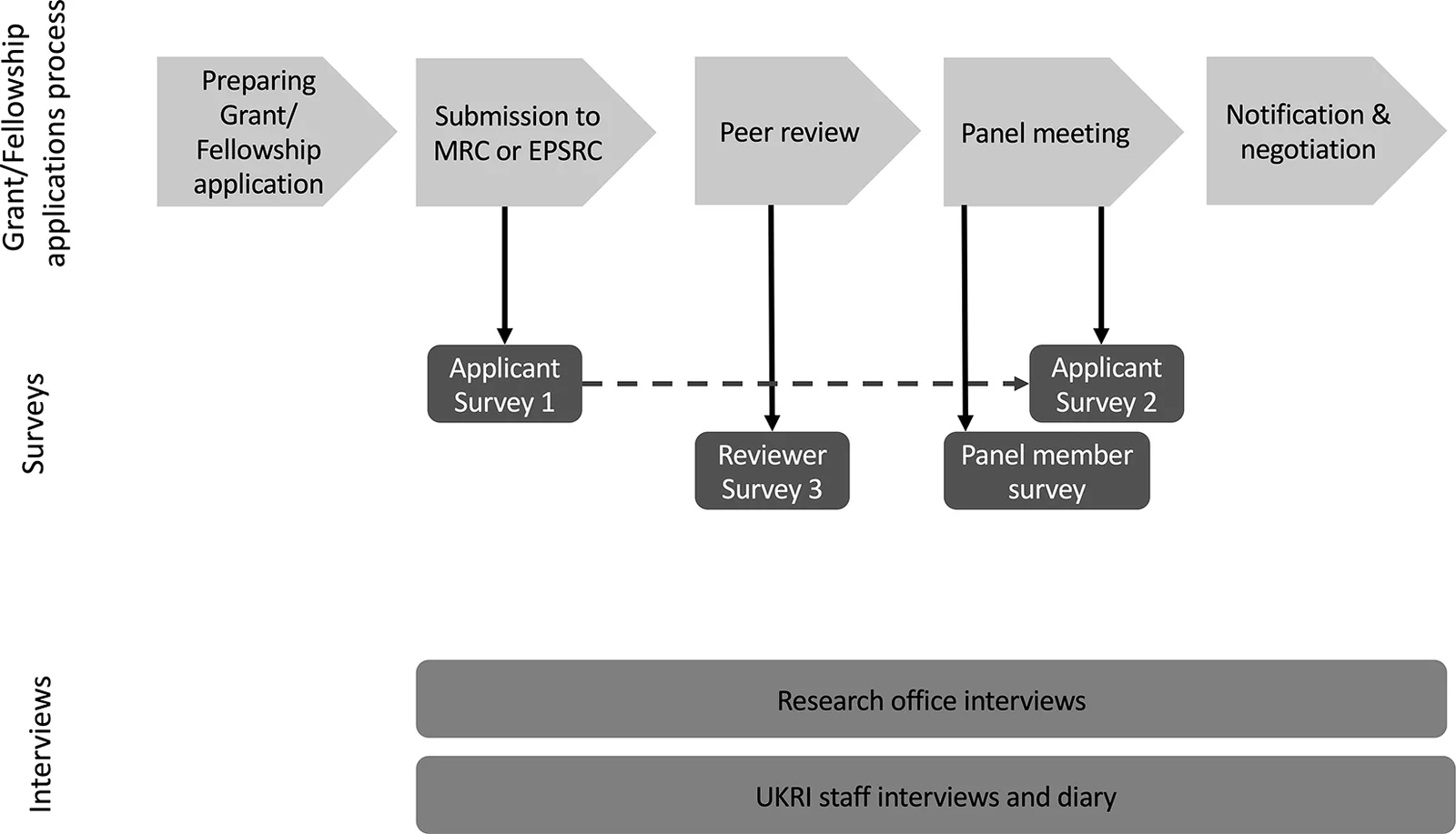
Project schema for data collection.

The main source of primary data was a series of four different surveys (administered by Ipsos), two aimed at research grant applicants, one for peer reviewers and one for panel members. The sequence of these is shown in Figure 1.

The process for developing each of the four survey instruments consisted of:

• Developing process maps for each of the four schemes above;
• Initial interviews with programme staff at the MRC and EPSRC;
• Review of surveys used in previous studies as summarised in Section 2;
• Development of draft surveys and testing through a series of cognitive interviews;
• Finalisation of survey, coding in online platform and final quality assurance.

The final drafts of the four surveys are provided in the Supplementary Material (Annexes B, C, D, and E), including an invitation to participants that noted the aims of the study, the data protection protocols we adopted, and approval from King’s College London’s Ethics Committee (approval number MRA-20/21–22938). All participants were adults and took part in the study voluntarily. Prior to participation, individuals were provided with an information sheet outlining the purpose of the study, what participation involved, data protection arrangements, and their right to withdraw at any time. Informed consent for participation in the surveys was obtained electronically and was implied by the completion and submission of the survey. Written consent was not collected because the surveys were administered online, involved minimal risk, and did not collect identifying personal data.

The surveys were designed to be relatively quick to complete, with the first applicant survey taking ten mins, the second five mins and the peer review and panel surveys five minutes each.

### Data analysis of survey responses

The data files from Ipsos were reviewed, and a number of quality assurance tests were conducted to check the face validity of the responses. This included running a series of box-whisker plots to estimate the amount of time taken (in days and hours) at various stages of the process and examining the distributions of job titles and other variables.

The main unit of analysis for estimating costs and transaction costs was grant/fellowship applications, but the information collected was from individuals (via surveys). This meant that responses had to be linked within and between different surveys. Each grant/fellowship could have a Principal Investigator (PI), a number of co-investigators (CI), and multiple reviewers. When estimating cost and transaction cost we treated PIs and CIs separately in our analysis, based on the assumption (subsequently borne out by our data) that PIs would – on average – spend more time on an application than CIs. (Note that panel responses were not linked as they were reported at the level of the panel meeting, but time/costs were estimated by calculating an average across all grants/fellowships considered by the panel).

As described in detail in Supplementary Materials Annex A, there were two main challenges in taking this approach. The first was the known missing data and the second was when there were conflicting responses for the same grant. For missing data, we estimated the average across the sample for that scheme for that specific survey. When there was more than one estimate of someone’s time, the self-reported or Principal Investigator figure was prioritized.

To monetize the time applicants, reviewers and panel members spent on the grant/fellowship, we commissioned the Higher Education Statistics Agency (HESA) for a data file that broke down salary costs by ‘Contract Level’ (i.e., job) and region. For each of the four job titles (professor, reader, senior lecturer, and lecturer), the average (mean) salary was used, with on-costs for National Insurance (a payroll tax) and pension contributions added. The costs were calculated at hourly rates for three scenarios. The first, following the approach adopted by universities in making TRAC submissions ^7^, used 1650 hours a year (=220 days × 7.5 h). The second, used in the sensitivity analysis (see Annex A), followed recent practice in estimating the costs for REF ( Technopolis, 2023), and used 1950 hours a year (= 260 days * 7.5 hours per day ^8^). A further sensitivity analysis used the TRAC 220 working days, but defined a day as 7 h (1540 h per year). This is consistent with the contracted hours of researchers in some universities and reflects the language of Survey 1, which asked respondents to report the number of 7-hour days spent.

The total and transaction costs were calculated for each of the grants/fellowships. The total cost is the sum of the cost to the applicants, peer reviewers, and panel members. Several sensitivity analyses were conducted, as reported in Annex A. The transaction cost was calculated by dividing the average cost of preparing an application by the average funding requested by the applicant, and then multiplying by the inverse of the scheme’s success rate (to account for failed applications). The success rates for the two specific MRC schemes were provided directly to us, and for the EPSRC were taken from UKRI’s website. ^9^

In addition to the sociodemographic characteristics of respondents, several questions surveyed their (i) experience of applying for research funding, peer review, and panel membership and (ii) views on whether the process was appropriate in terms of the time taken to prepare, review, or assess applications and how that compared to other funders. Each group of respondents was asked about the benefits of the process (beyond funding). A series of randomized statements developed from the existing literature were presented ( Box C), along with the opportunity to add any others. A sentiment index was created based on responses to the question on benefits in Applicant Survey 1.

Box C: **List of benefits, beyond funding, of applying for research funding.**

• Advances or fine-tunes my scientific, research or conceptual thinking
• Enables me to consolidate or organise my research plans
• Helps me generate new ideas that I wouldn’t have had otherwise
• Helps me plan the workflow for my research group
• Helps train/educate my graduate students and/or post docs
• Helps me develop new collaborations
• Helps me focus on the big picture rather than just the details of my projects
• Results in text or data that I can then reuse for future papers, grant applications, conference submissions, and other presentational materials
• Helps me gain experience in writing proposals
• Fulfils expectations of my role
• Brings me up to date with relevant literature
• Raises my awareness of topics of strategic interest to funders

Multivariable regression analysis was used to determine the cost drivers for Applicant Survey 1. We used a negative binomial regression model and reported exponentiated coefficients and 95% confidence intervals representing the ratio of the number of days spent preparing the application at different levels of the given predictor. The number of days rather than costs was used as the dependent variable because the costs calculated at least partially accounted for some of the predictors we wished to examine, such as job title and region. We only examined the results of Applicant Survey 1 (grant preparation), as this accounted for 76% of the total cost and had by far the largest sample.

Finally, the qualitative responses to the open-ended questions across the four surveys were analyzed, and common themes were identified through an iterative process. It should be noted that most of these questions focused on ways to improve the efficiency of the review process and thus had limited explanatory power for the quantitative analysis.

### Interviewing university research office staff

In addition to the four surveys, a small number of university staff (n = 21) were interviewed to understand additional contextual factors that may drive the costs and benefits of peer-review processes. The protocol for these interviews is provided in Supplementary Material Annex F. Semi-structured interviews were recorded and transcribed, and using an inductive approach, several salient themes were identified and tested through an iterative process.

For the qualitative interviews with university research office staff, informed consent was obtained verbally prior to the interview, including consent for audio recording. Verbal rather than written consent was used because the interviews were conducted remotely, involved minimal risk, and collecting written consent would have required the exchange or storage of identifying information, which the study sought to avoid. This consent procedure was reviewed and approved as part of the ethics approval. Ethical approval for the study, including all consent procedures, was granted by King’s College London’s Research Ethics Committee (approval number MRA-20/21–22938).

## 4. Results

As shown in Table 2, 12,617 people were surveyed between April 2022 and June 2023, with an overall response rate of 11%, which was broadly consistent across the four surveys (ranging from 8% for applicants in Survey 2 to 13% for reviewers). A total of 1330 responses made this one of the largest studies of its type to try to estimate the costs and transaction costs of peer-reviewed research grants and fellowship application processes. The anonymized data files for each of the four surveys are provided in Supplementary Materials, Annex G.

**Table 2.** Survey response rates.

Survey		Number
**Applicant Survey 1**	Surveyed	5148
	Responded	448
	Response rate	9%
**Applicant Survey 2**	Surveyed	2131
	Responded	178
	Response rate	8%
**Reviewer Survey 3**	Surveyed	4729
	Responded	628 *
	Response rate	13%
**Panelists Survey 4**	Surveyed	609
	Responded	76
	Response rate	12%
**Total**	Surveyed	12617
	Responded	1330
	Response rate	11%

* N = 1 participant dropped from the analysis due to incomplete data; analysis carried out using N = 627 participants

To determine whether our survey sample was representative, we compared the sociodemographic characteristics of the responders to Survey 1 to that of the applicant profile of the MRC and EPSRC for gender, ethnicity, and disability. As illustrated in Table 3, there is reasonable agreement for gender and disability but an overrepresentation of white respondents.

**Table 3.** Comparison of characteristics of Survey 1 and 2 responses to Research Council data.

Characteristic		Survey 1	Survey 2	Research council	
				MRC	EPSRC
Gender	Male	70%	78%	60%	76%
	Female	29%	22%	38%	22%
	Not disclosed	1%	0.6%	2%	2%
Ethnicity	White	80%	83%	68%	76%
	Non white	13%	13%	24%	18%
	Not disclosed	7%	4%	10%	7%
Disability	No	92%	92%	93%	91%
	Yes	2%	3%	2%	3%
	Not disclosed	5%	6%	5%	6%

The Research Council data are taken from UKRI EDI website for 2020/21: https://public.tableau.com/app/profile/uk.research.and.innovation.ukri./viz/EDIFundingdata2022forpublication/Awardratebyvalue

### Estimates of total cost and transaction costs for grant and fellowship applications

As illustrated in Table 4, the overall costs per grant/fellowship and transaction costs for the funding schemes surveyed were £24,445 and 13%, respectively. The total cost is the average total cost across each scheme, with scheme costs ranging from £17,202 for ESPRC Fellowships to £35,776 for the MRC DPFS, reflecting differences in the amount requested to some extent. The ratio of costs to the amount requested was 3% (with a consistent range between 2% and 3% across the four schemes). However, the success rates of the four schemes vary between 19% (EPSRC Fellowships) and 31% (MRC DPFS), thereby having a proportionately inverse impact on transaction costs. Across all four schemes, this was 13%, ranging from 7% (MRC DPFS) to 13% (EPSRC Standard Grants). Table 5 presents the distribution of costs by scheme and survey type. The primary observation from this table is that 89% of the costs fall on the applicants, with 7% on reviewers and the remaining 4% on panelists.

**Table 4.** Estimates of costs and transactions costs.

	MRC DPFS	MRC research grants	EPSRC standard research grants	EPSRC fellowships	Total (means)
**Applicant Survey 1**	£27,683	£18,185	£18,648	£9,957	£18,690
**Applicant Survey 2**	£5,503	£3,187	£2,779	£4,378	£3,051
**Reviewer Survey 3**	£1,658	£1,886	£1,746	£1,528	£1,793
**Panelists Survey 4**	£932	£1,631	£385	£1,339	£911
**Total cost (A)**	£35,776	£24,889	£23,558	£17,202	£24,445
**Total amount requested**	£80,112,988	£336,187,125	£233,567,352	£48,883,841	£698,751,305
**Average amount requested (B)**	£1,669,021	£903,729	£677,007	£842,825	£849,030
**Cost as % value of grant (A/B)**	2%	3%	3%	2%	3%
**Average success rate (C)** ^†^	31%	23%	26%	19%	23%
Transaction cost (A/B)*(1/C)	7%	12%	13%	11%	13%

^†^ *MRC data provided directly to us; EPSRC data taken from UKRI website.* https://public.tableau.com/app/profile/uk.research.and.innovation.ukri./viz/UKRICompetitiveFundingDecisions2022-23/CompetitiveFundingDecisions

**Table 5.** Distribution of costs by survey and scheme.

	MRC DPFS	MRC research grants	EPSRC standard research grants	EPSRC fellowships	Total
** Applicant Survey 1**	77%	73%	79%	58%	76%
**Applicant Survey 2**	15%	13%	12%	25%	12%
**Applicant Surveys 1 & 2**	93%	86%	91%	83%	89%
**Reviewer Survey 3**	5%	8%	7%	9%	7%
**Panelists Survey 4**	3%	7%	2%	8%	4%

### Time allocated to different tasks in preparing a grant or fellowship application

When asking respondents to allocate the time they spent on their grant or fellowship applications to various tasks, we provided three ‘other’ options in addition to the eight predefined tasks in Applicant Surveys 1 and four in Applicant Survey 2. Respondents were asked to state what the other task was, and then estimate the proportion of time they spent on that task. Overall, 39 of 470 (8%) respondents in Applicant Survey 1 chose this option and provided 39 suggestions, and 21 of 178 (12%) did so in Applicant Survey 2, providing 26 suggestions. These codes were reviewed and reallocated to the existing options or new codes generated, as listed in Box D. (The predefined codes can be found in the surveys in Supplementary Material Annexes B and C). Three-quarters of the total time was taken up by five tasks: preparing the case for support (30%); background reading and preliminary data analysis (14%); developing the research design (14%); preparing the budget, including internal administration and sign-off (9%); and preparing the justification of resources, data management plans, and other elements of the application beyond the case for support and budget (9%). Generating letters of support accounted for only 4% of the time.

Box D: **New tasks identified by applicant survey respondents.**

Applicant Survey 1

• Familiarising yourself with UKRI documentation and requirements
• Background reading and preliminary data analysis
• Engaging with collaborators/partners (including NHS Trusts)
• Developing the research design
• Preparing the case for support
• Preparing the budget, including internal administration and sign-off
• Preparing justification for resources, data management plans, and other elements of the application beyond the case for support and budget
• Generating letters of support from department heads or others

Applicant Survey 2

• Communications with university and partners
• Contacting UKRI
• Working on project and project plans

### Amount of time spent on preparing an application by various characteristics

Overall, the median total time spent preparing a grant/fellowship was 15 days. (Note here that we focus solely on Applicant Survey 1). This is illustrated in Table 6, which is broken down according to various characteristics. These crude data suggest that (unsurprisingly) PIs spend more time than CIs preparing grants/fellowships. Interestingly, those with high experience spend less time, along with male applicants and those who identify as white. There is an interesting relationship in the value of grants, with mid-sized grants taking longer. Finally, those that are funded spend more time preparing grants than those that are rejected.

**Table 6.** Median time spent preparing a grant/fellowship application by characteristics.

		Median number of days	Interquartile range		Number surveyed
			LQ	UQ	
** Respondents**	PIs	25	15	40	241
	CIs	6	3	15	207
	Total days	15	6	30	448
**Job title**	Professor	15	5	30	190
	Reader/Associate Professor	20	6	40	69
	Senior Lecturer	14.5	7	30	102
	Lecturer	20	6	31	87
**Experience** (i.e., how many grants/fellowships applicants submitted in past 3 years)	Low (1–3 submissions)	21	10	40	123
	Medium (4–7 submissions)	15	6	30	188
	High (8–47 submissions)	13	5	25	137
**Positive sentiment**	Yes	25	13	56	73
	No	15	5	30	375
**Negative sentiment**	Yes	20	7	30	70
	No	15	6	30	378
**Perceived Burden**	Very high	27	12	45	146
	High	15	6	30	240
	Neither high nor low	9	3	15	54
	Low/don’t know	2	1.5	3	8
**Comparative Burden**	Significantly more time	20	10	45	59
	More time	20	6	30	124
	About the same	15	5	30	221
	Less time	13	7	22.5	44
**Gender**	Male	15	5	30	314
	Female	20	8	35	130
	Not disclosed	17	3	40	4
**Ethnicity**	White	15	6	30	360
	Non white	25	10	48	58
	Not disclosed	11	4	23	30
**Disability**	No	18	6	30	413
	Yes	6	5	23	11
	Not disclosed	15	10	30	24
**Region of Higher Education institution**	Rest of England	14	5	30	278
	London	16	7	35	58
	Scotland/Wales/Northern Ireland	21	10	35	112
**Scheme**	MRC DPFS	15	5	30	33
	MRC research grants	20	5	30	207
	EPSRC Standard Research Grants	15	7	30	189
	EPSRC Fellowships	28	20	30	19
**Value of grant/fellowship**	Q1	15	7	30	113
	Q2	20	8	30	111
	Q3	20	4.5	40	112
	Q4	15	6.5	40	112
**Outcome of grant/fellowship**	Funded	19.5	7	30	78
	Rejected	16	6	30	322
	In progress	10	5	20	24
	Withdrawn	20	13	30	24

Positive sentiment (>/=6 rated substantial benefit); Negative sentiment (>/=7 rated no benefit

When these data were modelled through a regression analysis, using negative binomial regression to compare the number of days between groups, being a CI compared with being a PI, having more experience of submitting grant applications in the last three years, rating the applications as having a high burden, having a positive sentiment towards the process, having a disability, being in the middle value size of grant, and being funded compared with rejected (in contrast to comparing the medians in the crude data) were associated with fewer days spent on the application. Being female and of non-white ethnicity were significantly associated with spending a greater number of days on the application than being male/not disclosed or white/not disclosed ethnicity. The full results of the regression analysis are provided in Supplementary Material, Annex A.

### Perceived and relative burden of applicants

In Surveys 1 and 2, applicants were asked how they would rate the overall burden of that stage of a grant or fellowship application. In Survey 1, 86% of the respondents rated the burden as ‘high’ or ‘very high.’ However, in Survey 2, only 27% rated the burden similarly, with 47% selecting ‘neither high nor low’ at this stage. In Survey 1, applicants were also asked how the burden compared to grant/fellowship applications of a similar scale for other research funders. Interestingly, exactly half thought it about the same, with a further 41% saying it was ‘Significantly more time, compared to other funders’ or ‘More time, compared to other funders’.

### The benefits of developing and writing grant and fellowship applications

**Figure 2.**
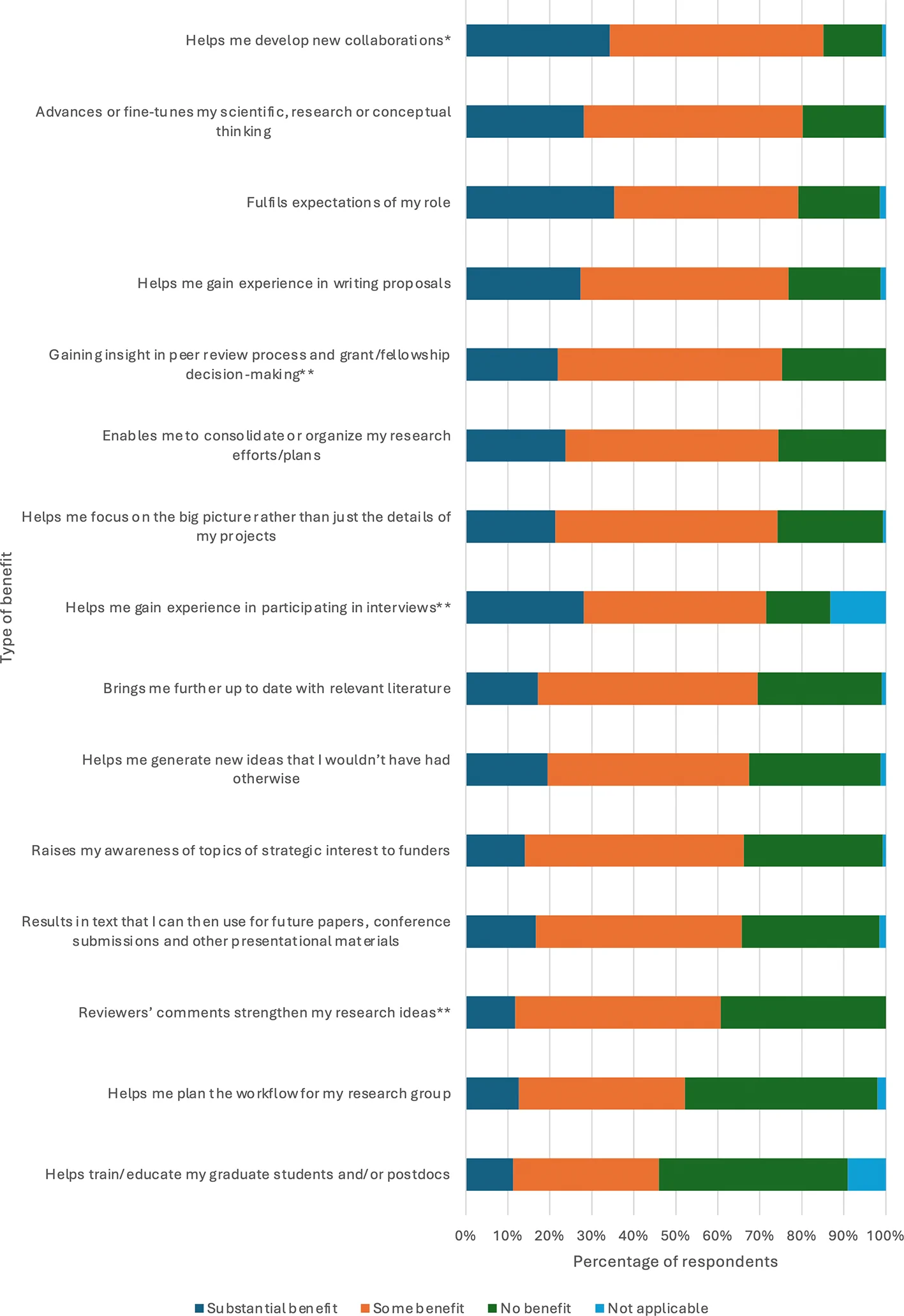
The perceived benefits of developing and writing grant and fellowship applications. * indicates only asked in Applicant Survey 1, and ** indicates only asked in Applicant Survey 2.

When presented with a closed list of potential benefits identified from the literature, 70% of applicants identified ‘substantial benefits’ or ‘some benefits’ across various statements. As illustrated in Figure 2, where the results from Applicants Surveys 1 and 2 have been combined, the greatest benefits identified were: ‘Helps me develop new collaborations’ (85% of respondents reporting substantial or some benefit), ‘Advances or fine tunes my scientific, research, or conceptual thinking’ (80%), and ‘Fulfils expectations of my role’ (79%). The areas of least benefit were: ‘Helps train/educate my graduate students and/or post docs’ (45%), ‘Helps me plan the workflow for my research group’ (52%), and ‘Reviewers’ comments strengthen my research ideas’ (61%).

### Qualitative feedback from applicants

There was considerable overlap in the type of response to open-ended questions on burdens and closing questions on ideas to reduce burdens and maximize the benefits of the application process. For example, the most salient theme in Applicant Survey 1 (where we had the largest number of responses) was to reduce the amount of information required in application forms (many of which were thought to be redundant), simplify forms, and standardize the process across UKRI. This was closely followed by suggestions for a triage process, where applicants only submit the case for support, and if it passes scientific peer review, then the research council seeks additional information. Another common response was that the overall success rates had to be increased, which meant increasing the total budget for research. In Applicant Survey 2, approximately one-third of those who added qualitative comments on the burden of the process highlighted the short timeframe for responding to comments, meaning that, while the overall burden between submitting an application and receiving a decision is relatively low, it is often concentrated in a one- to two-week period. Respondents suggested a longer time window for responding to reviews and establishing a clearer timeline at the outset, highlighting the impact of the concentrated burden on other responsibilities, both at work (e.g., teaching) and in their personal lives (e.g., childcare). Another notable theme in relation to reviews was the need for better quality or more constructive feedback. In Box E, some of the verbatim comments around these and the themes identified below are provided to contextualize this qualitative analysis.

Box E: **Selected comments around salient themes identified in the survey responses around ideas to reduce the burdens of grant and fellowship application processes.**

• “Ask for the case for support for the triage stage, and the data management, justifications and all the other ‘stuff’ only if the grant gets past that stage. Or even only if it is marked for potential funding as long as the extra documents are fine.”
• “Only ask for the case for support to be submitted with a ballpark cost. Then only successful applications give full costs, data management plans, etc., and assess these separately at this point.”
• “More funding needed, so success rates will be higher.”
• “Burdens would be substantially less if a higher percentage of good applications were funded.”
• “This particular grant was a less intensive proposal for me. I am much more often the PI, not the Co-I, and many of my proposals nowadays tend to be larger.”
• “A lot of the burden is not funder-generated, but rather caused by my own university and the lack of support it provides, and unnecessary red tape it requires me to go through, to submit the application.”
• “The overall burden is low but the one week deadline for responses concentrates all the burden into a very short period with no account for other responsibilities. The burden in that week is high.”
• “The timing of the responses meant that we had to work during school holidays and week-ends. The timing should take into account EDI considerations and workload - it is indirect discrimination.”
• “The time constraints were crazy last time - spent pretty much every free hour on it for two weeks. Wife (prof ) did the childcare and colleagues covered clinical sessions I swapped to free up time.”
• “The time burden is small, but recently reviews have become so bad that you learn nothing from them.”
• “The grant application process is wasteful of time and resource and stacked to favor a small cadre of researchers in ‘fashionable’ fields well represented on the respective funding boards. Other funders have considered a lottery as the most efficient way of disbursing grant funding, and I (and many other colleagues) am so disillusioned with UKRI’s application process that this now appears a far superior system.”

For the questions on burdens, one unique theme was the role of co-investigators, with a number noting that their contribution was significantly less than that of the Principal Investigator. The other salient theme unique to this question was the role of university administration in creating – in the views of the respondents – additional burdens in the process, either by having internal demand-management processes or by estimating budgets.

When asked about ideas for improving the process, a number of respondents proposed allowing the resubmission of failed applications, especially in the context of low success rates. Another salient topic was peer review, which had a number of aspects. For example, some felt the feedback of reviewers could be improved, which would make the process more beneficial to the applicant; others were concerned with biases in the peer review process and argued for either a fully blinded system or a much more transparent one; finally, there were a few comments suggesting greater dialogue between applicants and reviewers, akin to that used in journal peer review. Related to the issue of potential bias, several respondents suggested a lottery for the allocation of funding – i.e., random allocation among those considered ‘fundable’, given the marginal difference in their quality and low success rates.

Interestingly, compared to the open-ended question on burdens, the number of responses to a question on benefits was much lower (63 vs. 215), and nearly half of these commented that there were no benefits beyond the potential funding. For example, one respondent said,

*“There appear to be no benefits, other than (i) the slim chance of funding, and (ii) the fact that my role demands that I submit at least one grant application per year. The disbenefit is that a large portion of my time is spent writing grant applications instead of doing research.”*

It should also be noted that about 10% of the comments were very negative, illustrating the pressure individual applicants were under and, at the extreme, how that is impacting their mental health.

*“The idea that there is a significant benefit from a failed proposal relative to the time burden to prepare said proposal is ridiculous.”*

Despite this overall negative sentiment regarding the benefits of writing a grant or fellowship application, a number of positive comments and other benefits were identified. These included, for example, feedback for colleagues, learning the process of writing and submitting grant applications, managing time and expectations, and building collaborative networks.

### The reviewer perspective

The results of the reviewer surveys are summarized in Table 7. Overall, the median time taken by the reviewers was 5 hours, with little variation between characteristics. Reviewers were also asked how much time they spent on the MRC/EPSRC grant/fellowship application compared to other funders, with 78% saying it was about the same, 16% more than typical, and 5% less than typical.

**Table 7.** The time (in hours) reviewers took in assessing grant/fellowship applications, by various characteristics.

		Median number of hours	Interquartile range		Number surveyed
			LQ	UQ	
** Primary job title**	Professor	4	3	7	343
	Reader (or Associate Professor)	5	4	8	137
	Senior or Principal Lecturer	6	4	8	66
	Lecturer	6	4	10	50
	Lay reviewer	-	-	-	0
	Other	6	4	12	31
**Country of residence**	UK	4	3	7	398
	Other	6	4	8	229
**Experience** (i.e., how many grants/fellowships applicants reviewed in past 3 years)	Low	6	3	8	295
	Medium	5	3	7	189
	High	4	3	6	143
**Gender**	Male	5	3	8	439
	Female	5	3	8	179
	Not disclosed	4	4	6	9
**Ethnicity**	White	5	3	8	463
	Non white	6	4	10	98
	Not disclosed	5	4	8	66

**Figure 3.**
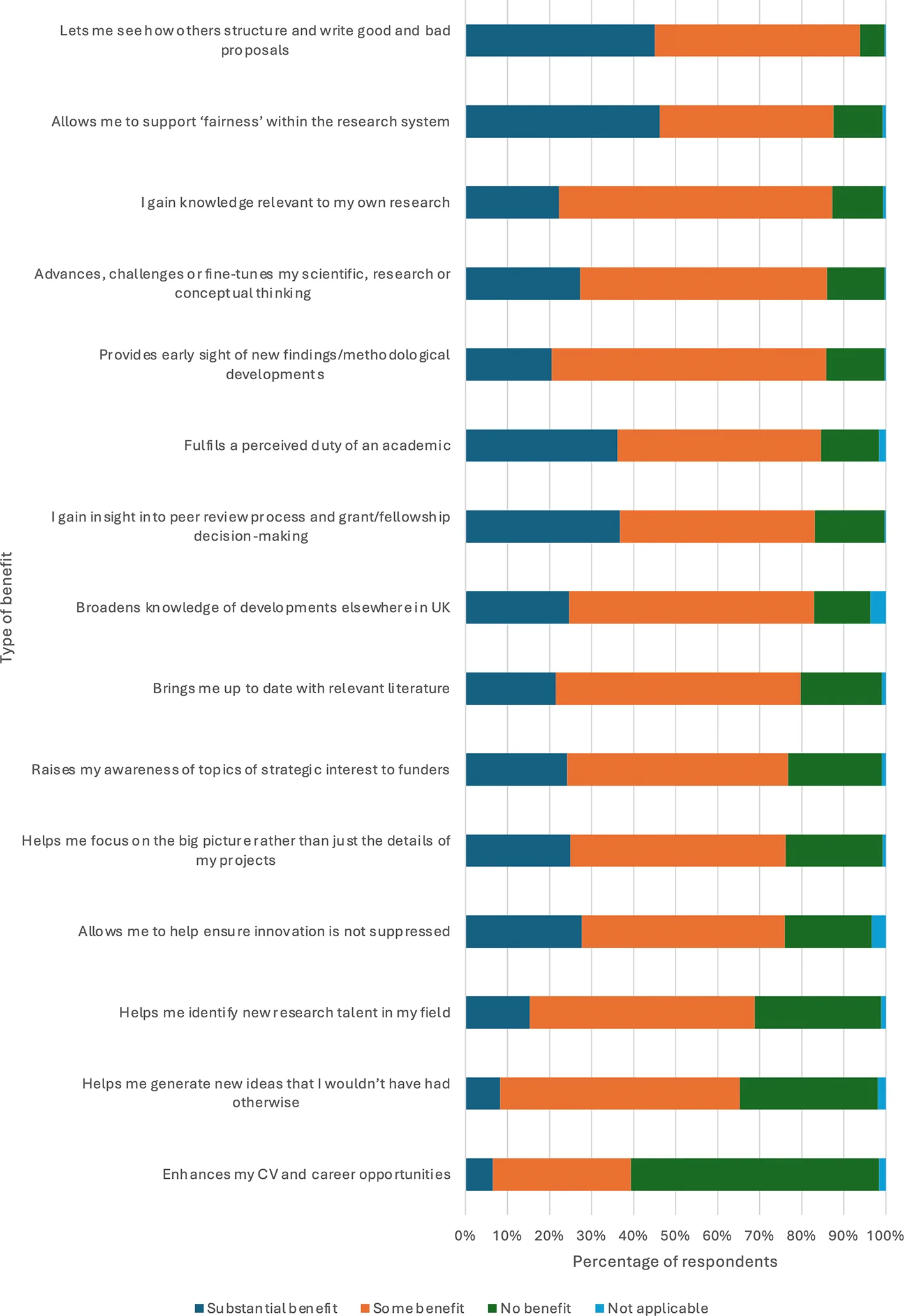
The perceived benefits of reviewing grant and fellowship applications.

When it came to benefits, perhaps not surprisingly, reviewers were on the whole more positive about the benefits of reviewing than applicants were about developing and writing proposals: across the various statements listed in Figure 3, 78% of reviewers identified substantial or some benefits (compared to 70% of applicants, Figure 2). Reviewers rated ‘Lets me see how others structure and write good and bad proposals’ as the highest (i.e., 94% of respondents rated it as providing a substantial or some benefit), followed by ‘Allows me to support ‘fairness’ within the research system’ (88%) and ‘I gain knowledge relevant to my own research’ (87%). The statement ‘Enhances my CV and career opportunities’ (39%) was the lowest, followed by ‘Helps me generate new ideas that I wouldn’t have had otherwise’ (65%) and ‘Helps me identify new research talent in my field’ (69%).

### The panelist perspective

Not surprisingly, the majority (66%) of the 76 panelists who responded to the survey were professors, with a further 9% readers. Overall, panelists estimated that they spent 33 hours preparing for a panel meeting and, on average, an additional 26 hours for interviews where applicable (i.e., for fellowships) (see Supplementary Materials Annex A for details). Of those panelists with comparative experience, 83% thought this was about normal and 17% more than typical.

**Figure 4.**
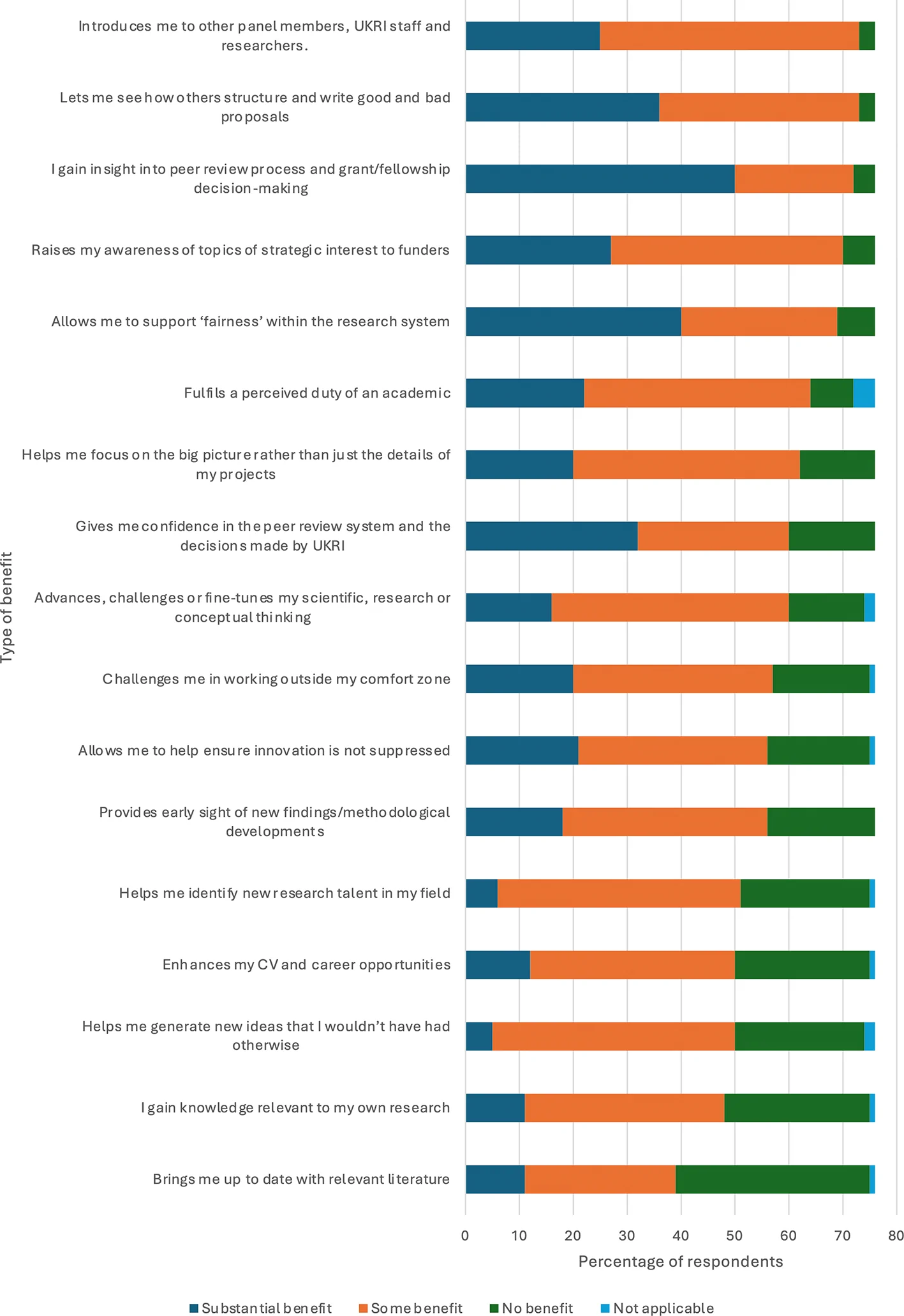
The perceived benefits of being a panelist.

As with the other surveys, the panelists were also asked about the benefits of being part of the process. As illustrated in Figure 4, over 95% of panelists considered ‘Introduces me to other panel members, UKRI staff and researchers’, ‘Lets me see how others structure and write good and bad proposals’ and ‘I gain insight into peer review process and grant/fellowship decision-making’ of being substantial or some benefit. The aspects that were reported least often as having substantial or some benefit were: ‘Brings me up to date with relevant literature’ (52%), ‘I gain knowledge relevant to my own research’ (63%), and ‘Enhances my CV and career opportunities’ (66%).

### The Research Office perspective

To gain an understanding of the administrative burdens and benefits associated with grant applications in universities, 21 Research Office staff members from 17 different universities were interviewed. The universities were selected to be broadly representative of the UK sector: eight of the 17 institutions (and 10 of the 21 interviewees) in the sample accounted for at least 50% of MRC and EPSRC applications between 2015–19; six institutions and eight interviewees for universities that accounted for between 50 ^th^ percentile and the 80 ^th^ of applications; and three institutions and three individuals from smaller, specialized, or teaching-focused institutions, accounting for the final 20 percentage point ‘tail’ of the distribution of applications by universities.

Just over half of the interviewees reported having specific expertise in EPSRC processes, while only three staff members reported having specific expertise with MRC. However, seven interviewees had expertise in more than one council, whether with the MRC and EPSRC (five staff members) or as a UKRI generalist (two). The majority of the interviewed staff members worked in Research Offices in universities that were part of a centralized support structure. Only one staff member reported being in a faculty-based research support role.

The research support roles of our interviewees were varied and comprehensive, covering every aspect of the ‘idea to award’ timeline – from training researchers in basic application and research skills, to idea development and horizon scanning, to application draft reviews, costings and agreements, submission, PI response, and preparation for mock interviews.

Of these areas of support, application review tasks (proofreading, application checking, reading for content, framing, etc.) were reported by most interviewees (18 of 21), while mock interviews and early application and idea development were identified as core parts of their role by under half of the interviewees’ responses (10 and 9, respectively). Other key tasks or activities included finance, costing, and approvals (11); internal demand management (9); training (8); and strategy and planning tasks, including research landscape scanning (8).

When asked to identify the most time-consuming part of their role, respondents provided a wide variety of answers, or, in the case of nine interviewees, did not address this point directly. Responses ranged from early application development (three interviewees); costings, contracts, or approvals (3); supporting academics on a one-to-one basis (2); internal peer review (2); and supporting internal peer review (2). Other individual responses included PI response tasks, mock interviews, checking ancillary documents such as data management plans, and demand management.

In contrast, when interviewees were asked to outline the key burdens of their roles – that is, their perception of their role and not a quantity or proportion of their time – a different set of responses emerged. Most notably, demand management processes (in which universities triage or filter applicants to UKRI schemes internally according to caps on applications set by the funder) were cited by nine interviewees as a significant burden. This part of the role was described as emotionally challenging and politically complex, a process for which HEIs are not set up, inordinately time-consuming, and duplicative of other processes within UKRI.

The discrepancies between what interviewees perceived as the most time-consuming burden and the burden they felt was significant in less-quantifiable ways may reflect the perceived value or importance of each of these activities. It is also worth noting that no interviewee reported having a formalized, quantitative, unit-cost tracking system for the time they spent on research development. Interviewees suggested that the reason for this is that the activities, burdens, and requirements for supporting and developing UKRI grant applications – and funded research more generally – are both too varied and difficult to quantify. Three interviewees specifically outlined the reasons such an approach would be unsuitable for research development. For all three, they believed fundamentally that the job of research development cannot be measured in terms of binary metrics of success or failure, precisely because their work feeds into a wider culture, environment, and support, which, in their view, should be provided regardless.

## 5. Discussion and implications

The headline results of our analysis – that the costs of peer review research grant and fellowship funding are approximately 13% of the value of funding applied for – are remarkably similar to those of previous studies ( Table 1). This is despite the use of different approaches. For example, not only is the current study one of the largest of its type, but it also followed grant/fellowship applications longitudinally and encompassed the cost of all the external actors in the process (i.e., applicants, reviewers, and panelists). However, our estimate does not include the operational costs of the research funder which, according to the latest accounts for UKRI, is around 3% of its total budget (recognizing that UKRI’s remit extends beyond grant funding). ^10^

Critically, this study not only looks at the transaction costs of peer review grant and fellowship funding but also looks at the cost drivers. For example, it is noteworthy that the vast majority of costs are borne by applicants (not the reviewers, panelists, or indeed the funder). When looking at the characteristics of the applicants, it is also notable that underrepresented groups, along with those with the least experience, are more likely to spend more time preparing a grant/fellowship application than other academic colleagues.

However, before drawing any policy implications from these findings, it is important to note the limitations of our study.

• **Transferability of findings to other funding schemes.** The project focused on four funding schemes from two research funders in the United Kingdom. Although the process of grant/fellowship peer review is similar across disciplines, funders, and regions, appropriate care should be taken when applying the results to other schemes. Success rates and funding amounts also vary by scheme.
• **Low response rates.** While the response rates were typical for this type of study, it is important to acknowledge that survey respondents may have a set of characteristics that are not representative of the broader population. As noted in Table 3, the sociodemographic characteristics of the sample and population were broadly similar; however, there may be other unknown biases in the data related to responding to the surveys.
• **Assumptions regarding missing data.** As noted in the Methods section (and Supplementary Materials Annex A), the need to use the grant/fellowship as the unit of analysis meant that we had to make a number of assumptions for missing data. These were tested in a sensitivity analysis and did not have a major impact.
• **Self-reported data.** As is inevitable in this type of study, the analysis relies on accurate and honest responses from the participants. Moreover, while we tried to make estimating time commitments as contemporaneous as possible, there will inevitably be recall errors and biases in relation to the time actually spent on various tasks.
• **The actual ‘value’ of peer review.** Although we surveyed respondents on the benefits of the peer review process, it is important to stress that conceptually what really matters is the ‘value added’ at each stage in decision-making – i.e., how decision-making is improved (in terms of funding the ‘best’ research) and how much ‘better’ the peer review process ultimately makes projects (whether funded or not). One approach to address this would be to apply contingent valuation methods to quantify and (potentially) monetize the benefits of peer review alongside qualitative exploration of the value added.

From a methodological viewpoint, although these limitations are real, it is difficult to come up with an alternative approach to estimating the cost of grant/fellowship peer review beyond ethnographic studies, which in themselves will either be very expensive or will focus on a smaller number of grants/fellowships.

While acknowledging these limitations, several policy implications can be drawn from this study:

1. **The focus of policy reform on research funders is misaligned.** As mentioned in the introduction, much of the contemporary policy focus is on funder reforms. However, the fact that 89% of the costs are to the applicants suggests that such a focus should be on universities. To illustrate this point, one of the commitments in the UK Tickell review on reducing research bureaucracy was “The requirement for letters of support will be removed, or if retained because it is a critical part of the assessment process, the information required will be significantly reduced.” As noted above, according to our results, this accounts for only 4% of applicant time.
2. **The ‘gold plating’ of grant and fellowship applications is likely to be driving up costs.** Hyper-competitive research culture in universities is likely to increase costs, partly driven by low success rates. As has been well documented, the narrowing of academic incentives on research grants and publications has had negative impacts on research cultures ( Edwards and Roy, 2017; Moran et al., 2020; Grant, 2021). However, without fundamental reform, academics will continue to apply for research grants in pursuit of reward and recognition within their universities and disciplines. This creates a vicious circle as it drives down funding rates (because of increased demand and/or reduced funding), thereby increasing competition. Consequently, academic researchers are likely to spend even more time ‘gold plating’ applications in an attempt to win funding (and advance their careers). One radical idea that could break this cycle is to offer a basic research income to all researchers who meet a set ‘quality’ criterion. If structured in a way that would be generous enough to allow support of substantive research activity, but at a threshold to incentivize the pooling of funding through collaborations, this could significantly reduce the need for and demand for extramural research support.
3. **Radical new approaches to funding could reduce costs.** Several funders have experimented with lotteries as a way to fund research. This is often motivated by both the need to reduce costs and to address concerns about biases in the peer review process. From an efficiency perspective, the key issue is defining the entry criteria and objectives of a lottery. If it is based on a short outline proposal that meets certain criteria, it may well reduce costs, as is being trialled by the British Academy for its small grant scheme. ^11^ However, if the aim is to make decisions at the margins of fundability, as the Swiss National Science Foundation does ( Heyard et al. 2022), then it is unlikely to reduce costs as a full application is still needed. Likewise, the use of artificial intelligence in peer review is often advocated as a way to reduce costs. For example, La Caixa Foundation has trialled an approach to remove from the peer review process outline proposals that are predicted not to be funded ( Cortes et al., 2024). However, while a number of funders are experimenting with the use of lotteries and AI, it should be noted that, to the best of our knowledge, evaluations of such interventions do not focus on the costs of the process.

In conclusion, this study estimated that the cost of grant and fellowship peer review funding processes is about 13% of the value of the grant, and that 89% of those costs are borne by the applicants. Concerns about the costs and benefits of the peer review process are legitimate, but the current policy debate needs to move from the idea of “not filling in unnecessary forms” (to requote former Prime Minister Johnson) to reducing jeopardy for academic researchers in applying for research grants.

## Data Availability

The quantitative survey datasets generated and analysed during this study, including all variables required to replicate the analyses and figures reported in the article (e.g., respondent characteristics, time estimates, cost calculations, and values underlying summary statistics and figures), are openly available via FigShare under a CC BY-SA 4.0 licence. See: Grant, Jonathan; Pollitt, Alexandra; Sreenan, Niall; Taylor, Clare (2026) *. Annex G Survey data. FigShare. Dataset.* *https://doi.org/10.6084/m9.figshare.31420586.v1* *.* Due to ethical and confidentiality considerations, the qualitative interview transcripts with university research office staff are not publicly available, as they contain potentially identifiable information. An anonymised summary of qualitative findings and the interview topic guide are included in the Supplementary Materials. Further access to de-identified qualitative data may be considered by the authors upon reasonable request, subject to approval by the King’s College London Research Ethics Committee and in accordance with participant consent.

## Footnotes

^1^

https://www.telegraph.co.uk/technology/2019/11/19/inside-innovate-uk-governments-innovation-agency/

^2^

*The Mark Gable Foundation* was written in 1948 and republished in Szilard’s 1961 memoirs *The Voice of the Dolphins and Other Stories.*

^3^

https://www.ukri.org/opportunity/developmental-pathway-funding-scheme/

^4^

https://www.ukri.org/councils/mrc/guidance-for-applicants/types-of-funding-we-offer/research-grant/

^5^

https://www.ukri.org/opportunity/epsrc-standard-research-grant/

^6^

https://www.ukri.org/what-we-do/developing-people-and-skills/epsrc/fellowships/an-overview-of-epsrc-fellowships/

^7^
The Transparent Approach to Costing (TRAC) is the methodology developed with the higher education sector to help them cost their activities. It is an activity-based costing system adapted to academic culture in a way which also meets the needs of the main public funders of higher education. See: https://www.trac.ac.uk/about/.

^8^
In the surveys a 7-hour day was specified, so this was adjusted for a 7.5 hour day to ensure consistency with previous costs estimates.

^9^

https://public.tableau.com/app/profile/uk.research.and.innovation.ukri./viz/UKRICompetitiveFundingDecisions2022-23/CompetitiveFundingDecisions

^10^

https://www.ukri.org/wp-content/uploads/2021/05/UKRI-270521-UKRI-Allocation-Explainer-2021-22-FINAL-PDF.pdf

^11^

https://www.thebritishacademy.ac.uk/news/promising-results-from-first-year-of-innovative-grant-awarding-trial .

## References

[ref1] Ayoubi C Pezzoni M Visentin F : The important thing is not to win, it is to take part: What if scientists benefit from participating in research grant competitions?. *Res. Policy.* 2019;48(1):84–97. 10.1016/j.respol.2018.07.021

[ref2] BEIS & DfE: Reducing bureaucratic burden in research, innovation and higher education. 2020. Reference Source

[ref3] Chalmers I Bracken MB Djulbegovic B : How to increase value and reduce waste when research priorities are set. *Lancet.* 2014;383:156–165. 10.1016/S0140-6736(13)62229-1 24411644

[ref4] Cortes CC Parra-Rojas C Perez-Lozano A : AI-assisted pre-screening of biomedical research proposals: ethical considerations and the pilot case of "la Caixa" Foundation. *Data & Policy.* 2024;6:e49. 10.1017/dap.2024.41

[ref5] Vrieze J de : Funders groan under growing review burden. *Science.* 2017;357:343–343. 10.1126/science.357.6349.343 28751589

[ref6] DSIT: Government Response to Independent Review of Research Bureaucracy. 2024. Reference Source

[ref7] Edwards MA Roy S : Academic research in the 21st century: Maintaining scientific integrity in a climate of perverse incentives and hypercompetition. *Environ. Eng. Sci.* 2017;34(1):51–61. 10.1089/ees.2016.0223 28115824 PMC5206685

[ref8] Gallo SA Thompson LA Schmaling KB : The Participation and Motivations of Grant Peer Reviewers: A Comprehensive Survey. *Sci. Eng. Ethics.* 2020;26:761–782. 10.1007/s11948-019-00123-1 31359327

[ref9] Gluckman P Ferguson M Glover A : *International Peer Review Expert Panel: A report to the Governing Council of the Canadian Institutes of Health Research.* Ontario: CIHR;2017.

[ref10] Gluckman PD : *Which science to fund: Time to review peer review?.* New Zealand Office of the Prime Minister's Science Advisory Committee;2012.

[ref11] Godlee F Jefferson T : *Peer Review in Health Sciences.* London: BMJ books;1999.

[ref12] Grant J : *Academic Incentives and Research Impact: Developing Reward and Recognition Systems to Better People’s Lives.* Washington DC: AcademyHealth;2021.

[ref13] Grant J Pollitt A Sreenan N : Annex G Survey data.Dataset. *figshare.* 2026. 10.6084/m9.figshare.31420586.v1

[ref14] Guthrie S : *Innovating in the Research Funding Process: Peer Review Alternatives and Adaptations.* Washington DC: AcademyHealth;2019.

[ref15] Guthrie S Ghiga I Wooding S : What do we know about grant peer review in the health sciences?. *F1000Res.* 2018;6:1335. 10.12688/f1000research.11917.2 PMC5883382 29707193

[ref16] Herbert DL Barnett AG Clarke P : On the time spent preparing grant proposals: an observational study of Australian researchers. *BMJ Open.* 2013;3(5):e002800. 10.1136/bmjopen-2013-002800 23793700 PMC3664356

[ref17] Heyard R Ott M Salanti G : Rethinking the Funding Line at the Swiss National Science Foundation: Bayesian Ranking and Lottery. *Stat Public Policy.* 2022;9(1):110–121.

[ref18] Hinrichs-Krapels S Grant J : Exploring the effectiveness, efficiency and equity (3e’s) of research and research impact assessment. *Palgrave Communications.* 2016;2:16090. 10.1057/palcomms.2016.90

[ref19] Irwin D Gallo SD Glisson SR : *Opinion: Learning from Peer Review. The grant-review process plays significant roles in the education of researchers and in shaping scientific progress.* The Scientist;2013. Reference Source

[ref20] Moran H Karlin L Lauchlan E : Understanding Research Culture: What researchers think about the culture they work in. *Wellcome Open Res.* 2020;5:201. 10.12688/wellcomeopenres.15832.1

[ref21] Morton J Shaxon L Greenland J : *Process Evaluation of the International Initiative for Impact Evaluation (2008–11). Triple Line Consulting.* London: Overseas Development Institute;2012.

[ref22] Research Councils UK: *Report of the Research Councils UK Efficiency and Effectiveness of Peer Review Project.* Swindon UK: Research Councils;2006.

[ref900] Robitaille JP Macaluso B Pollitt A Gunashekar S Larivière V : *Comparative scientometric assessment of the results of European Research Council funded projects. Bibliometric assessment report.* Brussels: European Commission, ERC Executive Agency;2015. Reference Source

[ref23] Rodriguez-Rincon D Feijao C Stevenson C : *Study on the proposal evaluation system for the EU R&I framework programme – Final report.* Brussels: Publications Office of the European Union;2022.

[ref24] Schroter S Groves T Højgaard L : Surveys of current status in biomedical science grant review: funding organizations ‘and grant reviewers' perspectives. *BMC Med.* 2010;8:62. 10.1186/1741-7015-8-62 20961441 PMC2974654

[ref25] Szilard L : *The Voice of the Dolphins and Other Stories.* New York: Simon and Schuster;1961.

[ref26] Technopolis: REF Accountability Review: Costs, benefits and burden Report by Technopolis to the four UK higher education funding bodies. 2015. Reference Source

[ref27] Technopolis: REF 2021 Cost evaluation. Final Report. 2023. Reference Source

[ref28] Tickell: Independent Review of Research Bureaucracy. Final Report. 2022. Reference Source

[ref29] Hippel T von Hippel C von : To Apply or Not to Apply: A Survey Analysis of Grant Writing Costs and Benefits. *PLoS ONE.* 2015;10(3):e0118494. 10.1371/journal.pone.0118494 25738742 PMC4349454

[ref30] Wessely S : Peer review of grant applications: what do we know?. *Lancet.* 1998;352(9133):301–305. 9690424 10.1016/S0140-6736(97)11129-1

[ref31] Weston J Gush J : *Evidence from ten years of research contract management.* Wellington: Royal Society of New Zealand;2012.

