# Supplementary material for: The costs and benefits of research grant funding peer review
Source: F1000Res. 2026 Jun 24;15:534. Originally published 2026 Apr 16. [Version 2] doi: 10.12688/f1000research.176989.2 (PMC13555001; doi:10.12688/f1000research.176989.2)
Supplement: Peer review history

# Reviewer report: Reviewer response for version 2

**DOI:** 10.5256/f1000research.203905.r497871

**Published:** 2026-08-11

**Authors:** Axel Philipps

**Affiliations:** 1 Martin Luther University Halle Wittenberg, Halle (Saale), Germany

This paper presents an impressive, methodologically robust study of research funding efficiency. The study is based on two major UK funding organizations and four of their funding schemes. The authors examine the costs associated with developing, writing, reviewing, and approving research proposals. To this end, they surveyed applicants, reviewers, panel members, and research officers involved in the funding process. Additionally, the authors discuss possible strategies for reducing the burden and cost of grant application and review procedures.

 The findings support previous research documenting the substantial burden that grant and fellowship applications place on researchers. Applicants invest considerable time and effort developing, writing, and refining proposals to maximize their chances of securing funding. In this regard, the authors contribute valuable information to the literature on transaction costs by estimating the monetary costs associated with grant and fellowship application processes. Furthermore, the paper provides an interesting and informative overview of the perceptions of applicants, reviewers, panel members, and research officers regarding the benefits of proposal review processes.

 Overall, the study offers important insights into the efficiency of research funding systems and how costs (and benefits) are distributed among those involved. However, the discussion and implications sections could be strengthened in several respects. Specifically, the paper could more deeply engage with the broader implications of the reported costs, further develop the relationship between costs and benefits, and elaborate on the policy implications of the results.

**1. Broadening the discussion of costs and funding efficiency**

 The paper primarily focuses on monetary costs and reports that preparing and writing proposals is equivalent to approximately “13% of the value of the grant” (Abstract). This finding could be discussed in greater depth. Research funding processes inevitably incur costs for reviewers, panel members, and administrative staff, but researchers applying for funding bear the largest burden. While some researchers are fortunate to have time allotted for grant writing in their workload, this is often not the case.

 Therefore, the authors could address a more fundamental question: Who ultimately pays for proposal preparation? Funding organizations do not compensate applicants for the time spent developing proposals. Instead, researchers typically absorb these costs themselves. In practice, this often means reducing the time available for teaching, research activities, and administrative responsibilities. It may also come at the expense of personal and family time, as previous studies (e.g., Herbert et al. 2014) have documented. Consequently, understanding the costs of proposal development and research funding efficiency requires a broader perspective than monetary estimates alone can provide. While monetizing these costs is valuable, it focuses attention on financial measures only and risks overlooking the personal, professional, and opportunity costs applicants incur. The authors should at least mention this risk.

**2. The significance of low success rates and repeated applications**

 A second concern is that the headline finding that the average cost of preparing, reviewing, and deciding on a proposal is equivalent to 13% of the value of THE grant (see Abstract) could be misleading. As this result is presented so prominently, it may give quick readers the mistaken impression that preparing a funded grant costs 13% of its value. However, the calculation includes unsuccessful applications.

 As the authors note, applicants typically submit multiple proposals before securing funding. The success rates across the funding schemes examined in the study range from 19% to 31%. This indicates that researchers generally have only a limited chance of obtaining funding with any one proposal. In other words, many applicants must prepare three or four proposals before securing support for a single research project. While some researchers may be more successful, many will need to invest substantial effort across several unsuccessful rounds of applications.

 This broader effort is largely invisible in the statement that “the cost of grant and fellowship application processes is 13% of the value of the grant” (Abstract). This finding does not fully convey how often researchers must prepare proposals before they can begin conducting their research.

 The authors break down the process of preparing a proposal into several tasks, including preparing the case for support, conducting background reading and preliminary data analysis, developing the research design, preparing budgets and internal approvals, and developing management plans (p. 11). While these activities are part of the research preparation process, the reality of grant competition is often different. In the best-case scenario, researchers repeatedly revise and repackage similar ideas, reorganize arguments, consult additional literature, and discuss multiple versions of an application over several rounds before eventually receiving funding. In the worst-case scenario, funding opportunities begin to shape research agendas. Researchers may have many viable, scientifically valuable ideas but focus only on those that receive funding.

 From this perspective, the true burden of externally funded research lies not merely in preparing individual proposals, but also in repeatedly and strategically adapting research ideas to highly competitive funding environments. Consequently, research funding is arguably more costly than the 13% figure suggests.

**3. Benefits are identified but not incorporated into a cost-benefit assessment**

 The surveys examine the perceived benefits of grant review processes, described as the study's primary objective in the Methods section (p. 6). Accordingly, the paper presents respondents’ views on the various functions and benefits of peer review. These findings are interesting and informative. However, the results are not fully utilized to answer the broader question raised earlier in the paper: How can the benefits of peer review be monetized and compared with the associated costs?

 While the benefits are summarized and discussed in detail, they are not integrated into a systematic comparison of monetized benefits and costs. The authors argue that such an approach would enable a cost-benefit assessment (p. 4); however, the paper ultimately does not provide one. If a full cost-benefit analysis was beyond the scope of the study, then it should have been excluded. Alternatively, the discussion section could offer reflections on how the benefits of peer review might be measured or monetized in future research.

**4. Implications for policy reform and the role of universities**

 Finally, the policy implications of the findings could be further developed. The paper argues that policy reforms are often misaligned when they focus primarily on research funders. This argument requires further clarification.

 While administrative work may account for only 4% of total costs, reducing early-stage administrative requirements could still lower the burden on applicants. Beyond simplifying administrative procedures, it is unclear what changes the authors consider most promising. Since applicants bear 89% of the costs, the discussion should more directly address which measures could reduce this burden.

 Similarly, the statement that universities play a large role in addressing the problem requires further explanation. What exactly should universities change, and how are these recommendations linked to the empirical findings? While universities may increase pressure on faculty members through performance expectations and incentives related to grant acquisition, universities are not solely responsible for the "hyper-competitive research culture" mentioned by the authors (p. 21).

 Hyper-competition and low funding success rates are characteristics of the broader scientific system, not just of universities. These characteristics are perpetuated by funding structures, evaluation criteria, and national research policies that extend beyond the university level. Therefore, meaningful reductions in proposal-related burdens would require institutional reforms and political willingness to fundamentally redesign the research funding infrastructure.

Is the work clearly and accurately presented and does it cite the current literature?

Yes

If applicable, is the statistical analysis and its interpretation appropriate?

Yes

Are all the source data underlying the results available to ensure full reproducibility?

Yes

Is the study design appropriate and is the work technically sound?

Yes

Are the conclusions drawn adequately supported by the results?

Partly

Are sufficient details of methods and analysis provided to allow replication by others?

Yes

Reviewer Expertise:

science studies, peer review, grant proposals, randomized grant allocation

I confirm that I have read this submission and believe that I have an appropriate level of expertise to confirm that it is of an acceptable scientific standard, however I have significant reservations, as outlined above.

## References

[1] : The impact of funding deadlines on personal workloads, stress and family relationships: a qualitative study of Australian researchers. *BMJ Open* .2014;4(3) : 10.1136/bmjopen-2013-004462 10.1136/bmjopen-2013-004462 PMC397576024682577

# Reviewer report: Reviewer response for version 2

**DOI:** 10.5256/f1000research.203905.r499842

**Published:** 2026-07-25

**Authors:** Alejandra Recio Saucedo

**Affiliations:** 1 National Institute for Health and Care Research Coordinating Centre, Southampton, UK

Reviewing ***The costs and benefits of research grant funding peer review *** by Pollit A, et al, has been a fascinating read that I truly enjoyed. In their study, the team quantifies the costs of preparing grants/fund applications as well as the costs of making a decision through peer review based on time spent on specific tasks. The methodology adopted is robust and the information provided in both the main paper and the supplementary data support reproducibility. It is also highly positive that the researchers add a note of the possibility to request some of the study data for secondary analyses (provided a justifiable reason is presented). Whilst I list some suggestions for improvement, I also took the opportunity of this review to add insights drawn from the paper’s contribution to the research funding landscape.

1. A conclusion in the abstract reads: “this would suggest that policies seeking to improve the efficiency of grant funding processes should consider the role of the university and research institute practices alongside funder requirements and assessment processes.” Since the Tickell review already highlights this consideration (e.g., pp 11;19;33), there is room to refine that statement and provide a recommendation that builds on the findings of the study in light of those published recommendations.
2. In the Introduction, the authors write that the key objective of the study is: “to develop estimates for the cost and transaction costs – the ratio of cost to funding – for a sample of different funding schemes administered by two funders, and to break down those cost estimates by various characteristics and ‘cost drivers’”. However, this one key objective is followed by a second objective: to explore the perceived benefit of peer review to applicants, reviewers and panellists. Although the paper builds on the reasons for this additional objective, the flow of the narrative to a. Develop an approach to quantify the costs of writing grant/fund applications and b. Understand the benefits of peer review, is not always linear. For example, keeping these two objectives in mind, the title of the paper becomes unclear given that “the costs and benefits of research grant funding peer review” did not seem to be the primary nor the secondary objective of the study. The objective of the study as presented in the title becomes evident on page 6 “The primary objective of the project was to estimate the monetized costs of the peer review process for a sample of research grant or fellowship schemes, and to balance that against the value derived from the process for the applicants, reviewers, panel members, and Research Council staff.” Still, also on page 6, another statement introducing an objective reads: “The focus of the study was on researchers making grant or fellowship applications to … ” This comment does not highlight an issue with the study design or the results presented, but the ambiguity in the scope of the study can be resolved by providing a full picture of the goals of the study, and how they interconnect, earlier in the paper.
3. The methods section was leaving me with a number of questions that were answered when looking at the materials published on FigShare. Given the amount of detail required to have a full picture of how the study was conducted and in light of the size of the paper, I suggest adding a statement within the methods suggesting reading that section alongside the supplementary materials.
4. Adding a note about scalability of the study would provide additional value to this work. Would researchers recommend replicating their methodology with other funders? on other programmes within the same funder? Is there data in the funding ecosystem that can be reused to calculate estimated costs?  

Following these specific notes on improvements, below are personal insights from a broader reflection of the study findings and how these may be used to make recommendations or invite discussions for positive disruption of our current research funding ecosystem.

1. Estimating costs of writing grant/funding applications, reviewing applications and making funding decisions based on self-reported time spent on specific tasks, provides a partial picture of the full cost of these activities, especially when administrative costs of staff in universities as well as staff in funding organisations are excluded. Given the limitations of the study (rightly pointed out by the researchers), it could be beneficial to add how the findings of the study may be used to design experiments aimed at improving (or redrawing) the programmes involved in the study. A recent publication would support the design of those practical experiments. 
2. There are recurring, seemingly unsolvable problems linked to research funding that I have heard for years: peer review is the "least worst" system we have to ensure research quality (so we focus our efforts in making the imperfect better); eliminating unnecessary bureaucracy will unlock innovation, increase efficiencies, reduce research costs, and accelerate science delivery and implementation (ensuring transparency and accountability however require some bureaucratic processes and therefore there is not much room for innovation); and the holy grail of solving all grant/funding challenges is to find the right balance between asking for the most minimal information from researchers and having enough information (funders) to ensure spending of public funds is traceable and reportable, all supported by a system that ensures decisions are fair and the best and most needed research is funded. I am convinced that it is time to re-draw the research funding sector not by trying to find a one-solution that fits all, but by providing space for organisations in the research sector to draw innovative and multiple ways of distributing research funds to fit different needs (e.g., a process for large infrastructure grants; a different process for fellowships; approaches that adopt random allocation and others that follow different stages of peer review due to the size of budgets or risks involved. Importantly, quantifying the full benefits of writing grants to draw a complete picture of the benefits that an agile research sector brings should underpin all processes. Implementing measures that allow for cost estimates from the start would enable the system to be monitored and improved without relying on proxies that are inevitably limited.

Is the work clearly and accurately presented and does it cite the current literature?

Partly

If applicable, is the statistical analysis and its interpretation appropriate?

Yes

Are all the source data underlying the results available to ensure full reproducibility?

Yes

Is the study design appropriate and is the work technically sound?

Yes

Are the conclusions drawn adequately supported by the results?

Yes

Are sufficient details of methods and analysis provided to allow replication by others?

Yes

Reviewer Expertise:

Research on Research

I confirm that I have read this submission and believe that I have an appropriate level of expertise to confirm that it is of an acceptable scientific standard.

## References

[1] Independent review of research bureaucracy: final report authors.

[2] Fast and flexible research funding: a review (RoRI Working Paper No. 20) authors.

# Reviewer report: Reviewer response for version 1

**DOI:** 10.5256/f1000research.195126.r483400

**Published:** 2026-06-01

**Authors:** Balazs Aczel

**Affiliations:** 1 Eotvos Lorand University, Budapest, Hungary

There is not much to add to this study, as it is critically important, well executed, and properly presented. I was only impressed by the effort that must have been behind this project. Its publication is timely, and I wish that it makes a lasting impact. 

 Minor comments: 

 - p9. "made this one of the largest studies of this type" could be dropped. The article is strong enough without "gold-plating".

 - Not listing males before females would be a modern practice

 - For Fig 2-4, the number of responses would be informative to add.

 Excellent work! I hope it will inspire others to pursue similar projects.

Is the work clearly and accurately presented and does it cite the current literature?

Yes

If applicable, is the statistical analysis and its interpretation appropriate?

Yes

Are all the source data underlying the results available to ensure full reproducibility?

Yes

Is the study design appropriate and is the work technically sound?

Yes

Are the conclusions drawn adequately supported by the results?

Yes

Are sufficient details of methods and analysis provided to allow replication by others?

Yes

Reviewer Expertise:

metascience

I confirm that I have read this submission and believe that I have an appropriate level of expertise to confirm that it is of an acceptable scientific standard.

# Response

**Published:** 2026-06-13

**Authors:** Jonathan Grant

**Affiliations:** King Abdullah University of Science and Technology, Saudi Arabia

Thank you very much for your thoughtful and supportive review. 

 We greatly appreciate your positive comments regarding both the importance and execution of the study.

 We have addressed the points you raised in the revised manuscript. Specifically, we have removed the phrase “made this one of the largest studies of this type” on page 9, reordered references to avoid listing males before females, and added the number of responses to Figures 2–4 for additional clarity.

 Thank you again for your careful reading of the paper and your constructive suggestions.

# Reviewer report: Reviewer response for version 1

**DOI:** 10.5256/f1000research.195126.r483396

**Published:** 2026-05-19

**Authors:** Christopher Cotton

**Affiliations:** 1 Queen’s University, Ontario, Canada

**Summary**

 Pollitt and colleagues estimate the costs of grant peer review across four funding schemes at two UK funders, finding that costs amount to approximately 13% of funded value, with 89% borne by applicants. The empirical work is careful, the longitudinal design is unusual and valuable, and the findings converge with prior international estimates (RCUK, 2006; Herbert et al., 2013; Gluckman, 2012; Rodriguez-Rincon et al., 2022). This is a useful addition to the empirical literature on research funding efficiency.

 My concerns are with how time estimates are interpreted as costs and the policy implications drawn from those estimates.

**Main comments**

 1. Grant writing is partly research, not pure overhead. The cost calculation treats grant-writing time as displaced from research, but the activities applicants report spending most of their time on, including preparing the case for support, background reading and preliminary data analysis, developing the research design, and preparing budgets and justifications, are themselves research activities. They have to happen at some point in the lifecycle of any funded project; the question is whether they happen before or after the money arrives. To the extent that grant writing substitutes for planning and design work that would otherwise occur later, the cost calculation overstates the true resource burden. Costly early effort can substitute for effort needed later, or improve the capacity for stronger subsequent research.

 The paper's own benefits survey supports this reading. The 85% of applicants who report that the process helps develop collaborations, 80% that it advances scientific thinking, and 79% that it fulfills role expectations are reporting on activities that are constitutive of research, not separable benefits of an overhead process. Treating these as side effects to be weighed qualitatively against monetized costs misclassifies the activity.

 2. The application process also performs other beneficial functions that are not captured by the analysis. Deadlines convert intentions into projects. Collaboration requirements assemble teams that would not otherwise form. Budget and timeline specifications impose planning discipline. The counterfactual to the current system is not the same research with less paperwork; it is different research, with different collaborative structures and planning standards. The paper measures only the cost side. The output-side, including what research happens, how well-structured it is, and how well-collaborated, is not assessed.

 3. Costly application also performs a screening function. In allocation systems where applications exceed funding availability and review-process capacity, application costs can improve allocative efficiency by discouraging low-probability submissions, incentivizing researchers to self-select toward proposals they believe are most competitive, and focusing reviewer time on the most credible ideas. This is a recognized mechanism in journal submission (Cotton, 2013) and applies at least as much to grant funding. Unlike journal submission fees, the costs imposed on researchers during grant development are themselves valuable for the eventual research output. Efforts to reduce these costs may have unintended consequences for allocative efficiency. The potential of costs to help maintain funding agency capacity and information should at least be acknowledged. (Note that I am the author of the above citation; I include it for context and do not require citation in any revision.)

 4. The empirical work cannot establish what the optimal level of upfront cost is. Some share of what researchers spend their time on is genuinely productive, while other activities like funder-specific formatting, redundant documentation, internal university approvals, and boilerplate letters of support likely are not. Where to draw the line is a substantive question, but it is not one the current paper can answer. The studies in Table 1, including this one, measure total time and resources; they do not measure what share of time is investment versus wasted overhead, what would have been spent on similar activities later, what screening value the cost generates, or how output and quality would change under alternative arrangements. Without a better understanding of these factors, the paper cannot say whether 13% is too high, too low, or about right. I would not be surprised to learn that 13% is actually a modest value for upfront planning expenses in many fields: engineering design costs ahead of construction projects, clinical trial protocol development, and complex software projects, for example.

**Implications for the policy section**

 The policy implications drawn in the discussion outrun the empirical work. In particular:

- The argument that 89% of costs borne by applicants implies reform should refocus on universities does not follow. Applicant time is largely a response to funder-set requirements; reducing those requirements would reduce the share borne by applicants. The 89% statistic is an accounting fact about where time is spent, not a causal claim about what causes it.
- The 13% transaction cost figure is dominated mechanically by the 1/success-rate multiplier (between 3.2× and 5.3× across the four schemes). Most of the variation in transaction costs across schemes reflects success rate variation, not process efficiency. The implication that the largest available lever is funding generosity, not process reform, is consistent with the data but underweighted in the policy translation.
- The administrative and screening side of the funding process, including funder operations, internal review structures, and coordination among funders, has received less empirical attention in this literature. A balanced policy conversation would examine both sides of the allocation process rather than focusing primarily on applicant burden. This is outside of the scope of the current study but could be acknowledged.
- The productive policy question is not whether 13% should be reduced but how to ensure the work captured by that figure is productive rather than wasted on adherence and overhead. These two questions point in different directions, and the empirical work in the paper does not distinguish them.

**Recommendation**

 The empirical work appears competent, and the findings merit inclusion in the literature on research system efficiency. But the policy conclusions are overstated. The discussion would be more useful if it walked through what can and cannot be concluded from the cost estimates, what the findings might suggest about reform, what would have to be true for those suggestions to hold, and what would still need to be established to actually support policy recommendations. As it stands, the discussion presents directional policy conclusions that depend on framing choices the empirical work itself does not establish — that grant-writing time is displaced from research, that benefits are separable from the activity rather than constitutive of it, and that the locus of measured costs is the appropriate target for reform. A revised discussion that surfaces these dependencies explicitly and that engages with the literature on how such costs could actually improve research outcomes would let readers evaluate the policy implications.

Is the work clearly and accurately presented and does it cite the current literature?

Yes

If applicable, is the statistical analysis and its interpretation appropriate?

Yes

Are all the source data underlying the results available to ensure full reproducibility?

Yes

Is the study design appropriate and is the work technically sound?

Yes

Are the conclusions drawn adequately supported by the results?

Partly

Are sufficient details of methods and analysis provided to allow replication by others?

Yes

Reviewer Expertise:

Economics, Science Policy, Metascience, Program Evaluation, Information Design

I confirm that I have read this submission and believe that I have an appropriate level of expertise to confirm that it is of an acceptable scientific standard, however I have significant reservations, as outlined above.

## References

[1] : Submission Fees and Response Times in Academic Publishing. *American Economic Review* .2013;103(1) : 10.1257/aer.103.1.501501-509 10.1257/aer.103.1.501

[2] : On the time spent preparing grant proposals: an observational study of Australian researchers. *BMJ Open* .2013;3(5) : 10.1136/bmjopen-2013-002800 10.1136/bmjopen-2013-002800 PMC366435623793700

[3] : The costs and benefits of research grant funding peer review. *F1000Research* .2026; 10.12688/f1000research.176989.1 10.12688/f1000research.176989.1 PMC1355500142723769

[4] Study on the proposal evaluation system for the EU R&I framework programme author.
